# Supplementary material for: Analyzing the comparative effectiveness of educational methods to foster children’s dengue knowledge
Source: PLoS One. 2026 Mar 10;21(3):e0341851. doi: 10.1371/journal.pone.0341851 (PMC12974831; doi:10.1371/journal.pone.0341851)
Supplement: Attachment — Submitted filename: Response to Reviewers.docx [file pone.0341851.s008.docx]

Dear Editor and reviewers,

Thank you for the detailed lecture on our manuscript, which has contributed significantly to improving it. You will find our answers below each of your comments, in blue.

**Comments to the Author**

1. Is the manuscript technically sound, and do the data support the conclusions?

The manuscript must describe a technically sound piece of scientific research with data that supports the conclusions. Experiments must have been conducted rigorously, with appropriate controls, replication, and sample sizes. The conclusions must be drawn appropriately based on the data presented.

Reviewer #1: Partly

Reviewer #2: Yes

2. Has the statistical analysis been performed appropriately and rigorously?

Reviewer #1: Yes

Reviewer #2: Yes

3. Have the authors made all data underlying the findings in their manuscript fully available?

The [PLOS Data policy](http://track.editorialmanager.com/CL0/http:%2F%2Fwww.plosone.org%2Fstatic%2Fpolicies.action%23sharing/1/010f0199093b19ec-82b14072-5956-4b0e-8a51-0d4c50136d52-000000/h1uQ-x8d2Vpx-reQu88UYYfHrf6qzPblOorxy3xRuRY=226) requires authors to make all data underlying the findings described in their manuscript fully available without restriction, with rare exception (please refer to the Data Availability Statement in the manuscript PDF file). The data should be provided as part of the manuscript or its supporting information, or deposited to a public repository. For example, in addition to summary statistics, the data points behind means, medians and variance measures should be available. If there are restrictions on publicly sharing data—e.g. participant privacy or use of data from a third party—those must be specified.

Reviewer #1: Yes

Reviewer #2: Yes

4. Is the manuscript presented in an intelligible fashion and written in standard English?

PLOS ONE does not copyedit accepted manuscripts, so the language in submitted articles must be clear, correct, and unambiguous. Any typographical or grammatical errors should be corrected at revision, so please note any specific errors here.

Reviewer #1: Yes

Reviewer #2: Yes

5. Review Comments to the Author

Please use the space provided to explain your answers to the questions above. You may also include additional comments for the author, including concerns about dual publication, research ethics, or publication ethics. (Please upload your review as an attachment if it exceeds 20,000 characters)

Reviewer #1: Title: Suggest to revise the title to reflect the overall aim of the study.

Following the reviewer's suggestion, we have changed the title “Direct instruction and workshops are equally effective in fostering children’s dengue knowledge” to: “Analyzing the comparative effectiveness of educational methods to foster children’s dengue knowledge.”

Introduction: Study justifications and objectives are clear.

Methods:
- What is the age of the repondents? Please justify the decision to select this age group.

Respondents are 10-year-olds. We included 10-year-old children because at this age they are literate, which allows both written knowledge assessments and workshops in which written resources can be used to work. In addition, the workshop material was pedagogically designed for 10-year-olds. All this information was added in the new version of the manuscript in the section “Participants”.

- Please specify what are the outcome measures studied. Why was it chosen? Any behaviour change outcome, apart from knowledge?

The outcome measure study is dengue knowledge. While recognizing the importance of behavior change for dengue prevention, we did not measure it because our study was aimed at comparing educational strategies in terms of their effectiveness to increase dengue knowledge (which is necessary, but not sufficient to produce behavioral change). On page 7, line 140 we added a sentence clarifying that “No behavioral change measure was included.”

Also, on page 14, lines, 293-295 the previous version clarified that further studies are needed to understand how knowledge translates into behavioral change. Now we moved that idea to lines 284-287, and included it as an essential limitation of our study that future studies should address.

Results:
- Table 2: What mean score is presented here? What is the distribution of the score?

The mean value presented in Table 2 refers to the mean of the total score grouped by time and experimental group. The “Measures” section as well as the table 2 note was expanded to clarify this.

State whether the scores were normal/not normally distributed.

The Score variable is a count measure constructed as the sum of all correct answers. Therefore, we did not expect raw values to necessarily follow a normal distribution, since count data are typically discrete, bounded at zero, and skewed. For this reason, we did not evaluate normality of the raw Score variable and, instead, went for a GLMM and tested normality of residuals among other diagnostic tests (GLMM does not assume normality of the dependent variable, but instead assumes normality of residuals). In order to provide more clarity, we added an explanation of model diagnostics in the “Analysis” section and added the GLMM residuals assumptions checks in the Supplementary data file.

- Table 3. Please state what is the reference group in the analysis.
In all models, the Talk group is the reference group. This was added in the table note.

Discussion and Conclussion:
- Will the results be applicable to other age groups?

This is a relevant question. Knowledge retention in the long term depends on long-term working memory systems. The neural basis of those systems relies on brain development, which occurs during the first two decades of life. Thus, the capacity to retain knowledge (i.e., to learn more dengue concepts) depends heavily on age during primary school. Therefore, we ignore whether our results will be valid for children at other developmental ages. Further studies are needed to check that. We have now stated that in the discussion section.

- How will the findings inform the planning and implementation of dengue health education in Argentina?

In order to answer this question, we have added lines 293-309 in the Discussion section.

Please revise these sentences to improve structure:
- Page 2; line 18

- Page 4; line 78, 79

Both sentences were corrected, thanks!

Reviewer #2: This is a well written manuscript on dengue education strategies targeting children age 10 years of age. The authors compared talk vs abbreviated workshop vs workshop and found no significant difference. Rather education and retention of specific concepts may depend more on the context.

Major
The abstract mentioned that children learned 3 dengue concepts and retained 2 however this is not expanded upon in the full manuscript.

Thanks for this comment. The data to conclude data is shown on Table 1. We have now expanded the explanation of this interpretation of the results in the “Discussion” section.

Line 49: Suggest amending the sentence. Reference 8 does not suggest that education is superior to legislation.

Thanks for this comment, the sentence was corrected.

Line 53: Qualify that this is the risk in the local context as different countries have different population at risk
Thanks for this comment. The sentence, as well as the reference, were deleted as they refer to Mexico but we do not have data on dengue prevalence by age groups in Argentina.

Minor

40-41 Arguably even if there is specific treatment for dengue, the most effective approach will still be prevention

41 Aedes aegypti is not the only vector for dengue
Right. To solve both minor comments, we have modified that sentence as follows: “The most effective approach to dengue is prevention, which involves controlling the virus's vector, that in the case of Argentina, is the *Aedes aegypti* mosquito [6].”
