# Supplementary material for: Analyzing the comparative effectiveness of educational methods to foster children’s dengue knowledge
Source: PLoS One. 2026 Mar 10;21(3):e0341851. doi: 10.1371/journal.pone.0341851 (PMC12974831; doi:10.1371/journal.pone.0341851)
Supplement: Attachment — Submitted filename: Response_to_Reviewers_auresp_2.docx [file pone.0341851.s009.docx]

Dear Editor and reviewers,

Thank you for the detailed lecture on our manuscript, which has contributed significantly to improving it. You will find our answers below each of your comments, in red.

Review Comments to the Author

Reviewer #3: A well-structured manuscript written with limited data. The results are encouraging in a way that children at their growing age can acquire knowledge about dengue and may translate it into basis of their behavior change, though not sufficient. Education and knowledge about dengue in early growing age, indeed, have potential to contribute in prevention of dengue transmission in dengue endemic regions, no matter which part of the world.
I have minor suggestions to improvise the manuscript:
- Check and simplify the sentences in lines 81-85

Thanks, sentences were corrected.

- Use of the words “direct instruction”, “Lecture” and “talks” are interchangeably used in the manuscript. I feel this has to be clearly mentioned in the introduction or in the methodology and more precisely use a single term throughout the text.

We agree. In this new version we used direct instruction across all the manuscript, only leaving “talk” in specific places, but not to refer to study groups.

- In line 153, “time x group” can be replaced with “time and group combined”

It was replaced

- In line 157, “ there was more than 80% overlap between the children who participated in each evaluation”, does this mean, there were children who did not take part in baseline survey but participated in T1 and T2 surveys? If yes, this need to be explicitly explained in the methodology.

Yes, we wanted to mean that. To clarify this point, we have added in the analysis section the following sentences:

“Moreover, given that GLMMs allow us to control unbalanced group sizes, there were children in T1 and T2 who did not take part in baseline, and vice versa, children in baseline that did not take part in T1 or T2. The percentage of children by group, at each time, that were also evaluated in the other two timepoints was calculated.”

Also, we have added in the Results section the following data: “(…) 17.34% of children in T1 and T2 not included in baseline”.

- In lines 168 and 170, What is “H” stands for? If it is “Kruskal–Wallis test statistic”, then this has to be mentioned in Analysis subsection under Methodology that you did the test to assess among the groups. The same comments and suggestions for F-statistics, as the values are shown in line 186.

To clarify this point, we have added the following sentences in the Analysis section:

“To assess the similarity of the different questionnaire versions in terms of scores and resolution times, we used a Kruskal-Wallis H test. The same test was implemented to compare the duration of the intervention activities and the number of participants included in them.”

And this at the end of the section we have added this sentence:

“The F-statistic was used for all GLMMs.”

- In lines 174-175, you mentioned “Likewise, after one month, boys and girls reduce their knowledge but do so in the same way in all three groups (p < 0.001)”. You assessed the gender effect on your outcome, need to be mentioned in the methodology section.

Thanks for alerting us about that mistake. We have not assessed the gender effect on our outcome as we wanted to check which intervention was most effective independently of gender. Thus, we have changed “boys and girls” by children in that sentence.

- Do you think “Incidence Rate Ratio” would be better option to explain the comparison between the groups than the“β” co-efficient in GLMM?

We thank the reviewer for the technical question. IRR as well as other exponential-based transformations could be a suitable option for informing the difference between the assessed groups. However, in the present study we decided to only inform the β for the sake of comparison with previous studies in similar populations in the country, in which the β estimate was informed.

Reviewer #4: (No Response)

Reviewer #5: Analyzing the comparative effectiveness of educational methods to foster children’s dengue knowledge

Well written/corrected manuscript. However, I have following suggestions.
1. Key words: Dengue is the topic but Dengue is not among the key words. If 6 words is limitation then remove Communicable Diseases and insert Dengue.

Thanks! Dengue was included as a keyword

2. Why the n (sample size decreased abruptly in T2 in comparison to base line and T1?

Baseline and T1 have slight variations in sample size due to the exclusion of unreliable questionnaires (protocols with scores below 5). Overall, sample size was maintained between Baseline and T1 because data was taken during the same day: as children were already in the school during the whole day, there was no attrition between those two measures. Instead, T2 was a measure taken one month later, in order to evaluate knowledge maintenance. At T2 various children were absent at schools during the days in which we collected the measures. Unfortunately, returning to schools some weeks later to complete data collection would have introduced other confusing variables given that knowledge retention is highly affected by time. Thus, in T2 we decided to keep only the data that was taken exactly one month later.

To clarify this point we added a note in Table 2.

3. Conclusion Line 308-309: However, few studies addressed the comparative effectiveness of educational interventions on dengue. Suggestion: Conclusion should include Authors findings only. Hence this line should be removed. But it might be placed in discussion section.

This sentence was removed

4. Line 309-311: Our study compared the most widely used educational approaches on this issue and demonstrated that direct instruction and workshop have similar effectiveness. Suggestion: If different approaches have similar effectiveness then please mention in a line, which method was cost effective, so that government/s may implement that in cost effective way.

Thanks for this important comment. We have added that line in the conclusion section.
